# Stabilizing Stable Coronary Artery Disease: No Beta-Blocker, No Problem?

**DOI:** 10.1016/j.jacadv.2024.101569

**Published:** 2025-01-17

**Authors:** Kuan-Yu Chi, Michael G. Nanna

**Affiliations:** a Department of Medicine, Jacobi Medical Center, Albert Einstein College of Medicine, Bronx, New York, USA; b Section of Cardiovascular Medicine, Yale School of Medicine, New Haven, Connecticut, USA

**Keywords:** beta-blocker, chronic coronary syndrome, percutaneous coronary intervention, stable coronary artery disease

**Figure:**
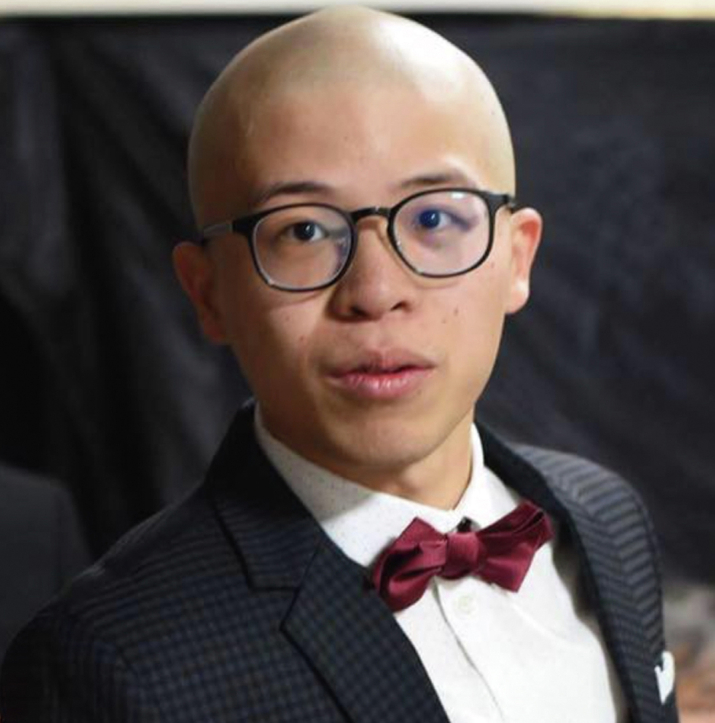

Shortly after joining Imperial Chemical Industries in 1958, Sir James Black developed propranolol in response to the sudden death of his father from a myocardial infarction (MI).^1^ Even after a Nobel Prize in 1988, few could have imagined the breadth of impact his discovery would have. Beta-blockers (BB) are now among the most commonly prescribed medications in the United States, with approximately one-tenth of patients in the United States receiving a BB in 2020^2^ for a broad spectrum of indications, including hypertension, heart failure with reduced ejection fraction, essential tremor, migraine, anxiety, portal hypertension, and coronary artery disease (CAD). Stable CAD represents a highly heterogenous population that can be broadly subdivided into 3 subgroups based on current literature^3^: 1) patients without a history of MI; 2) patients with a history of remote MI (≥1 year) without resultant complication, such as reduced ejection fraction (EF), heart failure, or recurrent MI; and 3) patients undergoing elective coronary revascularization. Evidence supporting BB use on stable CAD was largely extrapolated from studies on patients with MI, where their benefits were primarily established in the prereperfusion era.^4^ With the implementation of timely reperfusion therapy, potent antiplatelet agents, and statins,^5^ the role of BB inpatients with uncomplicated CAD has been challenged. Recent large well-conducted randomized trials have underscored this uncertainty,^6^^,^^7^ indicating no meaningful benefits of BB therapy for mortality or recurrent MI in patients post-MI with preserved EF. However, no large placebo-controlled trials have yet investigated contemporary BB use in patients with stable CAD. Thus, whether BB provide clinical benefits on mortality or cardiovascular (CV) outcomes beyond antianginal effects in this population remains unclear.

In that context, the work by Khan et al^8^ in this issue of *JACC: Advances* is both timely and valuable. The authors examine the effectiveness of incident BB initiated within 1 to 7 days after index percutaneous coronary intervention (PCI) for patients with stable CAD with preserved EF, aiming to address a critical gap in the literature. Using the TriNetX Database, they identified 132,242 patients with stable CAD with preserved EF who underwent PCI between 2009 and 2024, primarily through the International Classification of Diseases-10th Revision-Clinical Modification and Current Procedural Terminology codes. After propensity-score matching, the analysis eventually found that BB prescription was associated with a significant 11% increase in all-cause mortality, compared with no BB. There was no significant difference in hospitalization for MI, atrial fibrillation (AF)/flutter, and stroke. However, BB users were observed to have a significantly higher risk of hypotension, compared with nonusers. In the absence of randomized trials in this area, these findings reinforce the 2023 American College of Cardiology/American Heart Association guidelines on chronic coronary disease, which advise against routine BB use in patients without an EF below 50%, a history of MI, or other indications such as arrhythmia or hypertension.^3^

The authors should be applauded for employing rigorous methodology to conduct the analyses, working to combat inherent limitations of an observational design. First, the study was operated through a new-user design, excluding patients with a history of BB use prior to cohort entry to emulate a target trial, thereby minimizing prevalent user bias commonly encountered in retrospective analyses. Second, propensity-score matching was used to balance baseline characteristic between BB users and nonusers, reducing the potential impact of imbalance in measured confounders. Third, the authors utilized falsification endpoints and calculated E-values for the effect estimates of study endpoints to account for the potential impact of unmeasured confounding. These strategies highlight the authors’ efforts to rigorously assess the clinical effectiveness of BB in patients with stable CAD, collectively increasing the robustness of their findings. Notably, the finding of increased mortality with BB corroborates with a recent meta-analysis suggesting potential detrimental effects of post-MI BB in preserved EF patients.^5^ Additionally, a significant interaction between EF and BB for mortality benefits was recently identified in the U.S. PINNACLE (Practice Innovation and Clinical Excellence) Registry.^9^ However, even with meticulous methodology and supporting literature, attributing the observed mortality increase entirely to BB use is premature. Given the nearly identical rates of secondary CV endpoints (MI, stroke, and heart failure), the increased mortality in BB users is likely attributable to non-CV causes. While there was no significant difference in 2 falsification endpoints, a signal indicating an increased hazard of acute appendicitis with BB use (HR: 1.17; 95% CI: 0.95-1.45) suggests a potential imbalance of unmeasured confounders. This observation raises the possibility that residual unmeasured confounding contributed to the observed mortality difference despite the sophisticated statistical methods employed.^10^ As is often the case with comparative effectiveness research utilizing observational data, we are left with residual uncertainty.

Beyond the inherent limitations of the observational data set, the challenges posed by a population-level database lacking individual patient-level data must also be examined. Particularly, the absence of information on active disease status, medication use, and drug duration in the TriNetX database mandates cautious application of the results. For example, it is noteworthy that more than 40% of patients had a history of AF, yet none were on BB at baseline. While it is possible that some AF occurred in the context of acute illness which may not always require long-term rate control,^11^ this suggests a highly selected population. Similarly, the finding that over 80% of patients were on nitrates and/or calcium channel blockers before the index PCI indicates a deviation from typical practice patterns where BB are commonly used as a first-line antianginal treatment. Unfortunately, due to limitations intrinsic to the database, it remains unclear whether these prescribing patterns were driven by physician discretion or contraindications to BB use. Finally, the reliance on International Classification of Diseases-10th Revision and Current Procedural Terminology codes for diagnosing CAD and PCI precludes a detailed assessment of disease severity, coronary anatomy complexity, or the characteristics of the PCI performed. These factors must also be considered when interpreting the study findings and highlight the tradeoffs of comparative effectiveness research utilizing population-level databases.

As we navigate the seventh decade of BBs for the treatment of CAD, it is fair to question whether we are entering the “golden years” of beta-blockade? The present study by Khan et al undoubtedly offers valuable insights into the role of BB in patients with stable CAD with preserved EF undergoing elective PCI, suggesting that the uninspiring clinical benefits seen in contemporary patients with preserved EF following MI extend to those with stable CAD.^5^, ^6^, ^7^ However, we are left with as many questions as answers due to the observational design, underscoring the need for randomized clinical trials to provide definitive answers. Fortunately, 3 ongoing randomized trials—LIVEBETTER (NCT05786417), SMART DECISION (NCT04769362), and ABBREVIATE (NCT05081999)—are actively investigating BB use in patients with stable CAD. Awaiting the results of these trials, it is prudent to reconsider the routine use of BBs in patients with stable CAD with preserved EF who lack another clear indication for therapy. While it is too early to plan the retirement of BBs for stable CAD, scaling back to more targeted use is a logical first step. As the curtain rises on these pivotal trials, the stage is set to determine whether BBs will continue to play a starring role in stable CAD in the contemporary era or gracefully bow out as a relic of a bygone era.

## Funding support and author disclosures

Dr Nanna has done consulting for Heartflow, Merck, and Novo Nordisk; and has received research support from the 10.13039/100005485American College of Cardiology Foundation supported by the George F. and Ann Harris Bellows Foundation, the 10.13039/100006093Patient-Centered Outcomes Research Institute (PCORI), the Yale Claude D. Pepper Older Americans Independence Center (P30AG021342), and the 10.13039/100000049National Institute on Aging (K76AG088428). Dr Chi has reported that he has no relationships relevant to the contents of this paper to disclose.

## References

[1] Lyall J. (2010). James Black. BMJ.

[2] Database C.D. The top 200 drugs of 2020. Data from Medical Expenditure Panel Survey 2013-2020. https://clincalc.com/DrugStats/.

[3] Virani Salim S., Newby L.K., Arnold S.V. (2023). 2023 AHA/ACC/ACCP/ASPC/NLA/PCNA guideline for the management of patients with chronic coronary disease. J Am Coll Cardiol.

[4] Freemantle N., Cleland J., Young P., Mason J., Harrison J. (1999). Beta Blockade after myocardial infarction: systematic review and meta regression analysis. Br Med J.

[5] Chi K.-Y., Lee P.-L., Chowdhury I. (2024). Beta-blockers for secondary prevention following myocardial infarction in patients without reduced ejection fraction or heart failure: an updated meta-analysis. Eur J Prev Cardiol.

[6] Yndigegn T., Lindahl B., Mars K. (2024). Beta-blockers after myocardial infarction and preserved ejection fraction. N Engl J Med.

[7] Silvain J., Cayla G., Ferrari E. (2024). Beta-blocker interruption or continuation after myocardial infarction. N Engl J Med.

[8] Khan Safi U., Akbar U.A., Khan M.S. (2025). Beta-blockers after percutaneous coronary intervention for stable coronary artery disease and preserved left ventricular ejection fraction. JACC Adv.

[9] Arnold S.V., Silverman D.N., Gosch K. (2023). Beta-blocker use and heart failure outcomes in mildly reduced and preserved ejection fraction. JACC Heart Fail.

[10] Peri-Okonny P.A., Liu Y., Malaisrie S.C. (2019). Association of statin use and mortality after transcatheter aortic valve replacement. J Am Heart Assoc.

[11] Chyou J.Y., Barkoudah E., Dukes J.W. (2023). Atrial fibrillation occurring during acute hospitalization: a scientific statement from the American heart association. Circulation.

